# Associations between blood ethylene oxide levels and bone mineral density

**DOI:** 10.3389/fpubh.2025.1561920

**Published:** 2025-05-22

**Authors:** Wenwen Chen, Sujuan Lu, Min Lin, Kun Chen, Feng Huang

**Affiliations:** ^1^Shengli Clinical Medical College of Fujian Medical University, Fuzhou, China; ^2^Department of Geriatric Medicine, The First Affiliated Hospital of Wenzhou Medical University, Wenzhou, China; ^3^Department of Thoracic Surgery, The First Affiliated Hospital of Wenzhou Medical University, Zhejiang, China; ^4^Department of Geriatric Medicine, Fujian Provincial Hospital, Fuzhou, China

**Keywords:** ethylene oxide, bone mineral density, oxidative stress, inflammation, NHANES

## Abstract

**Background:**

Ethylene oxide (EO) is a toxic compound extensively used in industrial applications. This study quantified serum EO levels by measuring hemoglobin-bound ethylene oxide (HbEO). However, the link between bone mineral density (BMD) and HbEO levels remains unexplored.

**Methods:**

A total of 2,570 participants were evaluated using data from National Health and Nutrition Examination Survey (NHANES) (2015–2018). Generalized linear regression models (LRM) and restricted cubic spline (RCS) analyses were used to investigate the association between blood EO levels and BMD. Adjusted models were also applied for comprehensive analysis.

**Results:**

Blood EO levels and BMD were inversely related (*p* = 0.007). This RCS analysis also showed an L-shaped dose–response correlation between EO levels and BMD (*p* for nonlinearity <0.001).

**Conclusion:**

This study highlights a substantial correlation between EO exposure and BMD. Further randomized controlled trials are required to establish a causal relationship.

## Introduction

Ethylene oxide (EO) is a significant industrial and environmental chemical derived from ethylene, extensively used in sterilizing medical devices, producing various consumer products, and other industrial processes (1–3). Moreover, EO is also found in polluted air, vehicle emissions, and tobacco smoke (4, 5). At ambient temperature, EO exists as a gas, with inhalation serving as the primary route of human exposure. Once inhaled, EO is readily absorbed into the circulation, enabling widespread distribution and the formation of macromolecular adducts with nucleic acid and proteins (6).

EO-hemoglobin (Hb) adducts, specifically N-(2-hydroxyethyl) valine hemoglobin-bound ethylene oxide (HbEO), are frequently utilized as biomarkers for assessing exposure to EO After entering the bloodstream through respiration or dermal absorption, EO preferentially binds to hemoglobin to form HbEO adducts‌. The biological half-life of HbEO in the human body approximates the lifespan of red blood cells (approximately 120 days)‌ (7), theoretically reflecting average exposure levels over the past 2–4 months. This characteristic makes it superior to direct measurement of EO (half-life of mere minutes to hours) or its urinary metabolites‌ (8). Furthermore, the binding between EO and hemoglobin is irreversible‌. With continuous exposure, HbEO may gradually accumulate, rendering it more suitable for chronic exposure assessment‌. Previous studies have also demonstrated that HbEO has high sensitivity and effectiveness as a biomarker of EO exposure (9, 10). The International Agency for Research on Cancer regards EO as a group 1 human carcinogen (11). Previous studies have indicated that EO directly contributes to increased oxidative stress (OS). Furthermore, emerging evidence suggests a link between EO and various conditions, including elevated risks of developing asthma, diabetes, hypertension (HTN), cardiovascular disorders, and renal stones (12–17).

Healthy bones are essential for maintaining the body’s structural integrity, storing calcium, protecting vital organs, and anchoring muscles. Bone mineral density (BMD) is a widely used parameter for assessing bone health, with reductions in BMD serving as an early indicator of osteoporosis, a chronic condition that remarkably elevates the risk of fragility fractures (18). The prevalence of osteoporosis and associated fractures is rising sharply due to the aging population, leading to functional deterioration, reduced independence, economic and social burdens, and even death (19). Thus, the prevention of osteoporosis has emerged as a crucial challenge in modern medicine (20). Multiple factors contribute to the reduction of BMD, including environmental influences, genetic predisposition, and individual physiological factors such as endogenous hormone levels (19). Recent evidence suggests that environmental contaminants may significantly disrupt bone homeostasis (21, 22).

Besides, the association between HbEO and BMD remains poorly understood. Therefore, this study examined the possible correlation between blood HbEO levels and BMD by using data from the National Health and Nutrition Examination Survey (NHANES) to explore effective pathogenic mechanisms.

## Methods

### Data and participants

Data from NHANES between 2015 and 2018 were analyzed. This program, initiated by US Centers for Disease Control and Prevention, evaluated the health and nutrition of the US population. Data collection comprised stratification, multistage sampling, and probability cluster techniques. The National Center for Health Statistics Institutional Review Board approved the study protocols, and all individuals signed an informed consent form (23).

**Figure 1:**
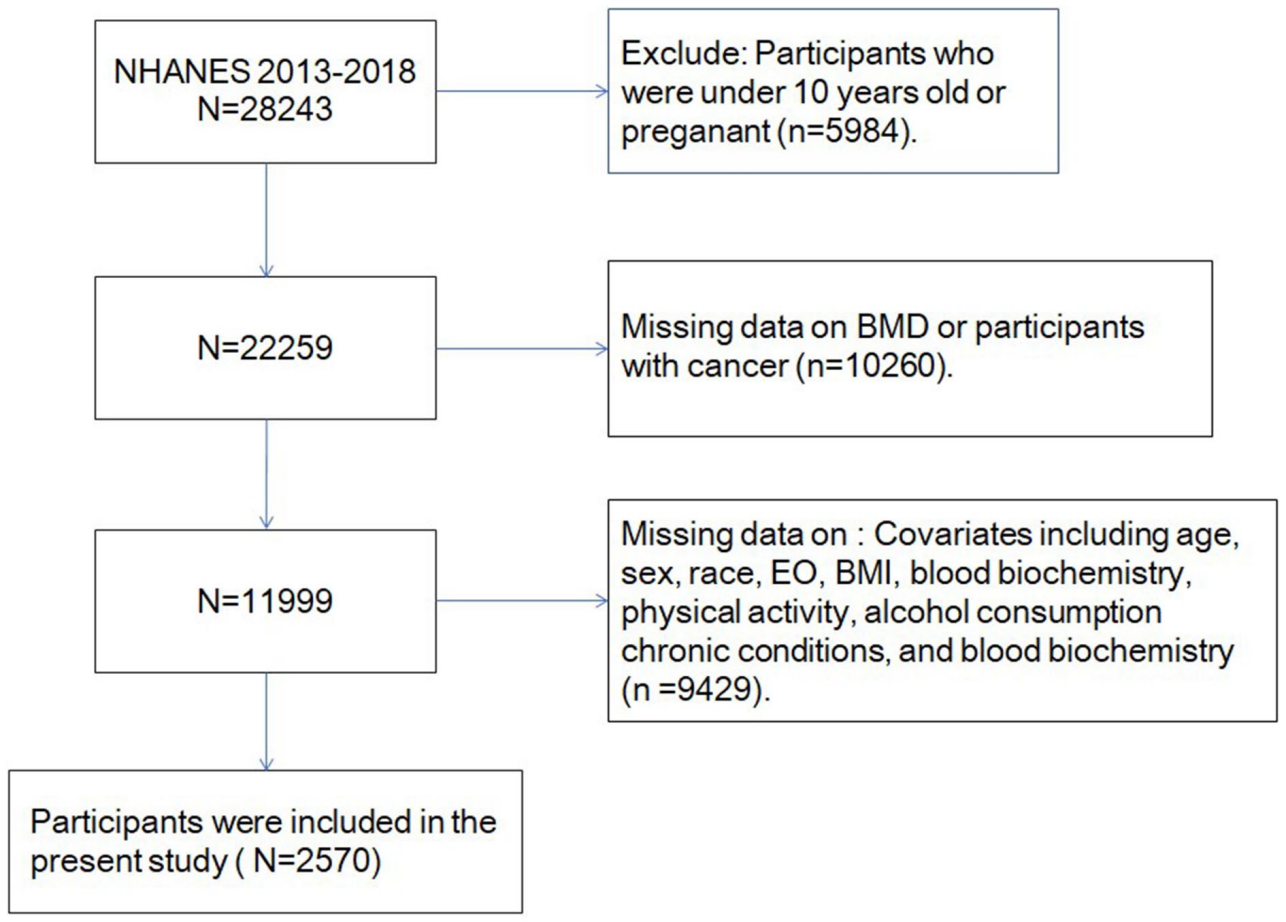
Flowchart portraying the sample selection.

Previous evidence suggested that most bone mass accumulation occurs in late adolescence (24) and adults face various adverse factors that can negatively affect their BMD (25). Participants were excluded as per the following criteria: (1) age < 10 years; (2) missing data on key variables, i.e., age, gender, race, total-body BMD, blood biochemistry, body mass index (BMI), physical activity levels, and alcohol consumption; and (3) previous malignancy [cancers often lead to bone loss (26)]. After excluding them, approximately 2,570 eligible individuals participated in the final analysis (Figure 1).

### Total bone mineral density

Bone mineral density (g/cm^2^) was observed *via* dual-energy X-ray absorptiometry (DXA) scans. Pregnant women, as well as individuals with a positive urine pregnancy test, were not included. However, individuals exceeding a BW of 136 kg or a height of 195.6 cm were deemed ineligible for DXA scanning. Whole-body DXA scans were carried out *via* a QDR 4500A fan-beam densitometer (Hologic, USA) as per the provided protocols. Scans were analyzed and reviewed by the Department of Radiology at the University of California, San Francisco, utilizing standard NCHS procedures.

### Exposure definitions

In this study, HbEO was used as a biomarker due to its remarkably high sensitivity in evaluating EO exposure (27). The measurement of HbEO adhered strictly to the guidelines outlined in the NHANES Laboratory/Medical Technologist Protocols Manual, available at https://wwwn.cdc.gov/Nchs/Nhanes/2013-2014/ETHOX_H.htm (accessed 25 June 2024). All specimen collection, processing, and transportation procedures were carried out per the standardized established procedures.

Total Hb levels and their adducts were quantified in this study. The modified Edman reaction measured Hb levels *via* a provided assay kit (Tech Diagnostics, USA). Moreover, HbEO levels in both whole blood and red cells were analyzed using high-performance liquid chromatography coupled with tandem mass spectrometry and are reported as pmol/g Hb. Detailed experimental procedures are provided in the NHANES manual.

### Covariates

The demographic variables evaluated in this study included age (in years), race (categorized as Black, White, or Other), sex (male or female), serum vitamin D level and dietary calcium intake. BMI was measured as the weight (kg)/height (m^2^) ratio. Medical conditions such as HTN, asthma, chronic kidney disease (CKD), diabetes mellitus (DM), and were self-reported based on previous diagnoses by physicians. Further, HTN was recognized if medication was taken for the condition, systolic blood pressure ≥140 mmHg, or diastolic blood pressure ≥ 90 mmHg. Levels of physical activity were quantified using the metabolic equivalent task (MET) and determined as: physical activity (MET·min/week) = suggested MET × exercise duration for respective activities (min/day) × frequency of exercise (days/week) (28).

### Statistical analysis

All data were statistically analyzed *via* the nhanesR package. Participants were stratified into 4 groups, with HbEO levels categorized by quartiles: Q1 (≤22.65), Q2 (22.65 < Q2 ≤ 32.7), Q3 (32.7 < Q3 ≤ 92.77), and Q4 (>92.77). The NHANES sample was designed to represent the population of the US, and analyses reported here are weighted following the NHANES Analytic Guidelines (29). Weighted chi-squared tests compared inter-groups of categorical variables. Because BMD is a continuous variable and confounding factors include multiple continuous and categorical variables, we use a weighted generalized linear regression model. These included model 1 (not adjusted) and model 2, with additional adjustments for race, age, sex, BMI, DM, HTN, CKD, asthma, MET, alcohol consumption, ALT, AST, Hb, HbA1c, serum vitamin D level and dietary calcium intake. Moreover, We investigated whether the shape of the relationship between BMD and HbEO was non-linear using the restricted cubic spline (RCS) regression model, and HbEO was included in the model as a continuous variable by using model 2. Subgroup analyses further explored possible sources of variability in this relationship. Several sensitivity analyses were conducted to evaluate the robustness of the findings. First, we used unweighted data to perform sensitivity analysis. Secondly, we converted HBEO into a continuous variable to determine if there is a linear relationship. Finally, explore the relationship between BMD of Lumbar spine and HbEO. In addition, we conducted variance inflation factor (VIF) analysis to evaluate multicollinearity and check the stability of the results. *p* < 0.05 was considered significant.

## Result

### Participant features

**Figure 2:**
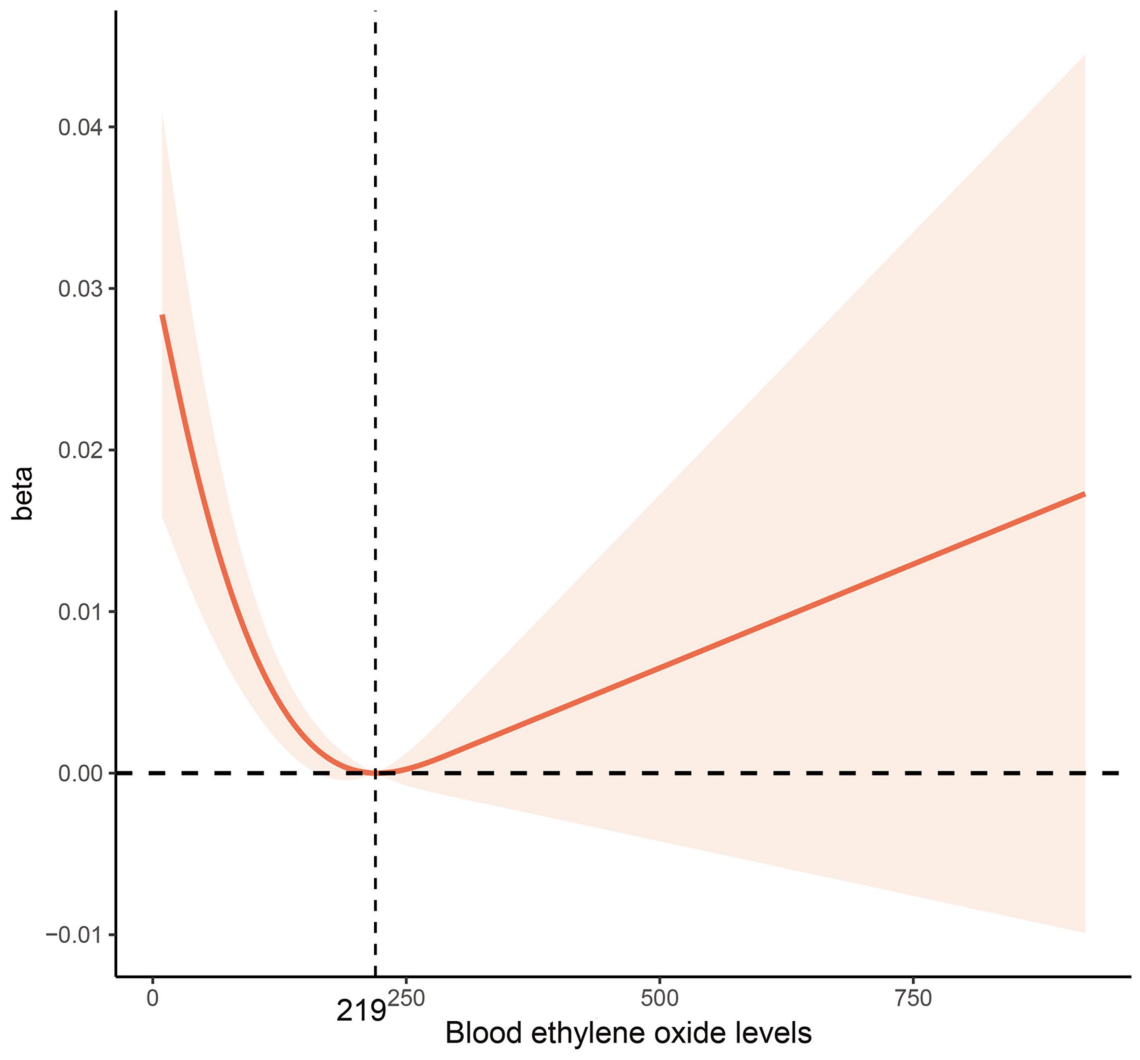
Multivariable-adjusted restricted cubic spline curve for the association between blood ethylene oxide levels and bone mineral density. The solid red line represents the fitted curve; the light red area represents the confidence interval.

Table 1 details the initial characteristics of all participants. All participants were American aged 10 to 59 (mean age, 35.104 ± 0.382), comprising 913 (39.35%) White and 1,382 (53.85%) male individuals. Table 1 shows the weighted features of the individuals arranged by HbEO quartiles. Moreover, substantial variations in confounding variables were observed across the quartiles (see Figure 2).

**Table 1:** Characteristics of the study population based on HbEO quartiles.

Variable	Total	Q1	Q2	Q3	Q4	P value
Age (years)	35.104(0.382)	35.907(0.704)	33.300(0.863)	31.811(0.657)	38.305(0.661)	< 0.0001
Race/ethnicity, *n* (%)						< 0.0001
Black	512(11.216)	101(7.857)	101(8.719)	128(12.761)	182(16.372)	
Other	1,145(27.844)	301(26.505)	341(33.151)	365(41.109)	138(14.493)	
White	913(60.940)	248(65.638)	199(58.130)	144(46.131)	322(69.135)	
BMI						0.43
<18.5	96(2.743)	15(2.027)	31(3.459)	31(2.817)	19(2.882)	
18.5 ≤ BMI < 25	780(29.536)	171(28.715)	185(26.187)	225(31.782)	199(31.909)	
25 ≤ BMI < 30	1,694(67.721)	464(69.258)	425(70.354)	381(65.401)	424(65.209)	
Sex, *n* (%)						0.005
Female	1,188(45.367)	342(50.217)	298(46.796)	304(44.805)	244(38.666)	
Male	1,382(54.633)	308(49.783)	343(53.204)	333(55.195)	398(61.334)	
TBMD (g/cm^2^)	1.113(0.004)	1.116(0.007)	1.114(0.005)	1.100(0.008)	1.119(0.006)	0.285
HbA1c (mmol/L)	5.420(0.022)	5.327(0.038)	5.395(0.028)	5.504(0.051)	5.492(0.044)	0.002
Alt (IU/L)	25.553(0.526)	24.000(0.603)	25.518(1.192)	26.414(1.012)	26.791(1.700)	0.138
Ast (IU/L)	25.164(0.417)	23.878(0.495)	24.920(0.667)	25.865(0.741)	26.399(1.395)	0.088
Hemoglobin (g/dl)	14.388(0.037)	14.146(0.059)	14.308(0.072)	14.292(0.064)	14.826(0.065)	< 0.0001
Serum vitamin D level (nmol/L)	65.784(1.106)	67.282(1.452)	64.837(1.529)	64.479(2.236)	65.853(1.519)	0.536
dietary calcium intake (mg)	1039.556(14.640)	1030.891(32.310)	1073.167(21.558)	1047.146(26.858)	1013.139(42.309)	0.5
Drinks(g/day)						< 0.0001
0	2010(72.222)	498(68.941)	546(80.796)	542(77.847)	424(63.971)	
0–500	273(13.087)	84(17.117)	55(11.076)	54(11.718)	80(11.157)	
> = 500	287(14.691)	68(13.942)	40(8.128)	41(10.435)	138(24.872)	
CKD, *n* (%)						0.509
No	2,331(91.846)	592(92.985)	581(91.931)	585(92.125)	573(90.194)	
Yes	239(8.154)	58(7.015)	60(8.069)	52(7.875)	69(9.806)	
Asthma, *n* (%)						0.243
No	2,154(83.257)	554(84.982)	549(84.407)	534(83.538)	517(79.918)	
Yes	416(16.743)	96(15.018)	92(15.593)	103(16.462)	125(20.082)	
Hypertension, *n* (%)						< 0.0001
No	2026(78.015)	506(79.063)	533(82.103)	540(83.928)	447(68.503)	
Yes	544(21.985)	144(20.937)	108(17.897)	97(16.072)	195(31.497)	
Diabetes mellitus, *n* (%)						0.114
DM	195(6.989)	44(5.615)	44(7.029)	52(7.990)	55(7.838)	
IFG	85(3.307)	28(4.535)	23(2.966)	7(1.297)	27(3.674)	
IGT	51(1.741)	7(0.911)	19(2.557)	14(2.080)	11(1.725)	
No	2,239(87.963)	571(88.938)	555(87.447)	564(88.633)	549(86.763)	
MET(met·min/week)						< 0.0001
<2000	1,072(40.862)	273(41.483)	291(44.236)	291(42.705)	217(35.607)	
2,000–5,999	747(28.600)	206(32.066)	198(29.263)	203(32.559)	140(20.837)	
> = 6,000	751(30.538)	171(26.451)	152(26.500)	143(24.736)	285(43.556)	

HbEO, hemoglobin-bound ethylene oxide; BMI, body mass index; TBMD, Total bone mineral density; CKD, chronic kidney disease; DM, diabetes mellitus; IFG, impaired fasting glucose; IGT, impaired glucose tolerance; HbA1c, glycated hemoglobin A1c; ALT, alanine aminotransferase; AST, aspartate aminotransferase. MET, metabolic equivalent task. Q1 ≤ 22.65; 22.65 < Q2 ≤ 32.7; 32.7 < Q3 ≤ 92.77; Q4 > 92.77.

### Relationship between EO and BMD

Table 2 presents the substantial correlation between the levels of HbEO and BMD. No significant link was seen between the two in Model 1 (*p* for trend = 0.921).

**Table 2:** Association of HbEO with TBMD.

	Q1	Q2	Q3	Q4	P for trend
β(95% CI)					
Model I	Ref	−0.00162(−0.01750,0.01426)	−0.01518(−0.03600,0.00565)	0.00313(−0.01188,0.01814)	0.921
Model II	Ref	0.00614(−0.00766, 0.01994)	−0.00946 (−0.02632, 0.00739)	−0.01944(−0.03382,-0.00507)	0.007

HbEO, hemoglobin-bound ethylene oxide; BMI, body mass index; TBMD, Total bone mineral density; COPD, chronic obstructive pulmonary disease; CKD, chronic kidney disease; DM, diabetes mellitus; IFG, impaired fasting glucose; IGT, impaired glucose tolerance; HbA1c, glycated hemoglobin A1c; ALT, alanine aminotransferase; AST, aspartate aminotransferase; MET, metabolic equivalent task. Model 1: unadjusted. Model 2: Adjusted for age, race, sex, BMI, DM, hypertension, CKD, asthma, MET, drinking status, ALT, AST, hemoglobin, and HbA1c, dietary calcium intake, vitamin D levels. Q1 ≤ 22.65; 22.65 < Q2 ≤ 32.7; 32.7 < Q3 ≤ 92.77; Q4 > 92.77.

Model 2 was constructed, incorporating additional variables, including age, race, sex, BMI, DM, HTN, CKD, asthma, MET, alcohol intake, ALT, AST, Hb, HbA1c, serum vitamin D level, and dietary calcium intake.

In the adjusted model 2, upregulation of HbEO was substantially correlated with reduced BMD (*p =* 0.007).

When using the first HbEO level as the reference, the *β* coefficient for level 2 was 0.00614 (95% CI: −0.00766, 0.01994), for level 3 was −0.00946 (95% CI: −0.02632, 0.00739), and for level 4 was −0.01944 (95% CI: −0.03382, −0.00507), with *p* for trend = 0.007. The multivariable-adjusted RCS model further examined the correlation between HbEO and BMD, revealing an L-shaped dose–response curve (*p* for nonlinearity <0.001). This model indicated that BMD reaches its minimum value when HbEO exceeds 219.56. The VIF values of all factors were less than 5 (Supplementary Table 4).

### Subgroup analyses

All parameters, i.e., gender, age, race, BMI, alcohol intake, HTN, CKD, DM, asthma, and MET, were used as stratification variables to assess the trend in effect size (Table 3).

**Table 3:** Association between HbEO and TBMD in subgroups.

Variable	Q1	Q2	p	Q3	p	Q4	p	p for trend (character2integer)	P for interaction
Age(years)									0.009
10–17	ref	0.019(−0.019, 0.057)	0.283	−0.003(−0.041, 0.035)	0.863	−0.029(−0.093, 0.035)	0.319	0.545	
18–29	ref	0.019(−0.005, 0.043)	0.116	0.008(−0.018, 0.034)	0.546	0.011(−0.018, 0.040)	0.429	0.533	
30–39	ref	−0.026(−0.057, 0.006)	0.103	−0.039(−0.065,-0.013)	0.005	−0.049(−0.073,-0.025)	<0.001	<0.001	
40–49	ref	0.002(−0.021, 0.025)	0.838	0.01(−0.015, 0.036)	0.403	−0.013(−0.038, 0.012)	0.284	0.4	
50–59	ref	0.028(−0.009, 0.064)	0.128	−0.014(−0.050, 0.023)	0.442	−0.04(−0.065,-0.014)	0.004	0.002	
Race/ethnicity									0.139
Black	ref	−0.009(−0.049, 0.031)	0.613	−0.011(−0.044, 0.022)	0.445	−0.005(−0.044, 0.033)	0.748	0.751	
Other	ref	−0.003(−0.018, 0.011)	0.626	−0.011(−0.028, 0.007)	0.212	0(−0.026, 0.026)	0.974	0.614	
White	ref	0.014(−0.008, 0.035)	0.188	−0.012(−0.041, 0.017)	0.389	−0.03(−0.047,-0.013)	0.002	0.001	
Sex									0.154
Female	ref	0.01(−0.010, 0.029)	0.309	−0.011(−0.035, 0.014)	0.372	−0.024(−0.045,-0.004)	0.024	0.024	
Male	ref	0.002(−0.018, 0.022)	0.813	−0.008(−0.029, 0.012)	0.401	−0.016(−0.035, 0.003)	0.090	0.054	
BMI									0.06
<18.5	ref	0.038(−0.014, 0.089)	0.141	0.018(−0.029, 0.064)	0.431	0.044(−0.063, 0.151)	0.397	0.547	
18.5 ≤ BMI < 25	ref	0.031(−0.001, 0.063)	0.060	−0.008(−0.034, 0.019)	0.548	−0.008(−0.033, 0.016)	0.479	0.222	
25 ≤ BMI < 30	ref	−0.003(−0.018, 0.011)	0.630	−0.013(−0.031, 0.005)	0.137	−0.029(−0.043,-0.015)	<0.001	<0.001	
Asthma									0.309
No	ref	0.009(−0.007, 0.024)	0.244	−0.011(−0.029, 0.007)	0.208	−0.015(−0.030,-0.001)	0.040	0.018	
Yes	ref	−0.006(−0.039, 0.027)	0.691	−0.015(−0.053, 0.022)	0.401	−0.044(−0.086,-0.001)	0.046	0.044	
Hypertension									0.041
No	ref	0(−0.018, 0.019)	0.968	−0.012(−0.031, 0.007)	0.207	−0.012(−0.029, 0.004)	0.135	0.083	
Yes	ref	0.026(−0.009, 0.062)	0.136	0(−0.024, 0.025)	0.968	−0.032(−0.057,-0.007)	0.016	0.005	
CKD									0.117
No	ref	0.004(−0.010, 0.018)	0.537	−0.011(−0.028, 0.006)	0.198	−0.023(−0.037,−0.010)	0.002	0.002	
Yes	ref	0.033(−0.016, 0.083)	0.165	0.003(−0.042, 0.048)	0.898	0.007(−0.036, 0.051)	0.713	0.952	
Drinks (g/day)									0.807
0	ref	0.008(−0.007, 0.023)	0.261	-0.01(−0.028, 0.008)	0.258	−0.021(−0.039,-0.003)	0.027	0.015	
0–500	ref	0.027(−0.024, 0.078)	0.269	0.007(−0.043, 0.057)	0.772	0(−0.042, 0.043)	0.981	0.936	
> = 500	ref	−0.024(−0.075, 0.026)	0.317	−0.022(−0.075, 0.031)	0.387	−0.042(−0.082,-0.002)	0.042	0.051	
MET(met·min/week)									0.664
<2,000	ref	0.018(−0.002, 0.038)	0.074	0.003(−0.019, 0.025)	0.776	−0.012(−0.034, 0.009)	0.246	0.197	
2,000–5,999	ref	0.001(−0.021, 0.024)	0.922	−0.013(−0.043, 0.017)	0.377	−0.021(−0.051, 0.008)	0.144	0.14	
> = 6,000	ref	0.001(−0.024, 0.025)	0.955	−0.021(−0.048, 0.007)	0.133	−0.022(−0.045, 0.002)	0.066	0.033	

HbEO: hemoglobin-bound ethylene oxide; BMI: body mass index; TBMD: Total bone mineral density;; CKD, chronic kidney disease; DM, diabetes mellitus; IFG, impaired fasting glucose; IGT, impaired glucose tolerance; HbA1c, glycated hemoglobin A1c; ALT, alanine aminotransferase; AST, aspartate aminotransferase. MET, metabolic equivalent task. Adjusted for age, race, sex, BMI, DM, hypertension, CKD, asthma, MET, drinking status, ALT, AST, hemoglobin, HbA1c, serum vitamin D level and dietary calcium intake. Q1 (≤22.65), Q2 (22.65 < Q2 ≤ 32.7), Q3 (32.7 < Q3 ≤ 92.77), and Q4 (>92.77).

Subgroup analysis revealed considerable associations between HbEO levels and BMD based on age (*p* for interaction = 0.009). Specifically, higher HbEO levels were related to lower BMD in individuals between ages 30 and 39 (*p* < 0.001) and 50 and 59 (*p* for trend = 0.002). Among individuals with HTP, those with the highest HbEO Q4 had a substantial negative relationship with BMD (*β* = −0.032; 95% CI: −0.057,-0.007; *p* for trend = 0.005, *p* for interaction = 0.041). No substantial variations were seen in the remaining subgroups (*p* > 0.05).

### Sensitivity analysis

The results of sensitivity analysis were consistent with those of main analysis. Details are listed in Supplementary Tables 1–3.

## Discussion

This cross-sectional study utilized NHANES data from 2015 to 2018, including 2,570 individuals who met the predefined inclusion criteria. According to our knowledge this analysis is the first to investigate links between exposure to EO and BMD in the US population. The univariate analysis did not find a significant correlation between the two variables. However, after adjusting for potential confounding variables, HbEO levels were inversely correlated with total-body BMD. Previously, it was reported that BMD is a vital parameter for assessing osteoporosis, with decreased BMD documented as constituting a key risk for osteoporosis-associated fractures (30). The identification of modifiable risk factors is highly important, as osteoporosis can be effectively prevented and managed before fractures develop. This study underscores the negative association between exposure to EO and the BMD in the normal population. Further randomized controlled trials are required to establish a causal relationship.

Environmental pollution is strongly associated with BMD. We did find a number of studies implicate various environmental pollutants with BMD (21). For example, Scimeca et al. (8) found that heavy metals such as cadmium, lead, chromium, mercury accumulation affects bone microarchitecture in osteoporotic patients. Particulate matter (PM), especially suspended particulate matter with a diameter ≤ 2.5 *μ* m in the air, is one of the core indicators of air quality monitoring, including acids, water droplets, elemental carbon, organic carbon, polycyclic aromatic hydrocarbons (PAHs), metal dust, mineral dust, etc. (31). Adami et al. (32) found that chronic inflammation caused by PM2.5 exposure may lead to the imbalance of bone resorption by osteoclasts and bone formation by osteoblasts. Tian et al. (33) found that PM2.5 exposure leads to ROS production and oxidative stress. These free radicals can cause cell damage, including bone cells. Endocrine disruptors such as bisphenol A (BPA), phthalates, and per-and polyfluoroalkyl substances (PFAS) compounds will Imitate or interfere with natural hormones like estrogen, which play a role in bone health (34). This disruption interferes with bone remodeling—the process of bone formation and resorption—thereby leading to reduced BMD (35).

EO, a reactive epoxide, is widely recognized as a significant threat to health and has been declared a human carcinogen by United States Environmental Protection Agency (36). The link between EO and malignancy remains a subject of ongoing debate. A recent study indicated an increased risk of mortality in lympho-hematopoietic cancers (37) although this was not confirmed by a meta-analysis (38). In terms of non-malignant diseases, research is relatively limited, although elevated risks of diabetes, HTN, and dyslipidemia have been described (39, 40). Wang et al. (41) found a substantial relationship between EO exposure and depression. Further, findings from a case–control study suggest that prolonged, low-dose EO exposure may adversely impact cognitive activity (42). Furthermore, previous research has demonstrated that prolonged exposure to EO adversely affects cognitive abilities and may contribute to the development of anxiety (43). The association between HbEO and BMD remains poorly understood. An animal experiment investigated the effect of ethylene oxide on the bone morphogenetic protein (BMP) bone induction ability in male mice, and found that ethylene oxide reduced BMP bone formation activity by about one-third (44). In addition, we did not find any previous records specifically examining an association between EO and BMD.

The precise biological processes responsible for the EO-BMD association are not clear. Inflammation and OS are hypothesized to affect the correlation between EO exposure and BMD. Bone is a metabolically active tissue that undergoes continuous remodeling, a tightly regulated physiological process involving the osteoclast resorption of aged bone and osteoblast-mediated formation of new bone (45). Disruption of this balance, characterized by increased osteoclast activity and insufficient osteoblast-mediated bone formation, may lead to progressive bone loss, increased fragility, and elevated fracture risk (46).

Previous research has demonstrated that both OS and inflammation contribute to the pathogenesis of osteoporosis by enhancing osteoclastic activity and inhibiting osteoblastic function (47–49). Growing evidence suggests that exposure to EO may elevate OS and inflammation. Experimental preclinical studies have reported that EO exposure is correlated with a reduction in the levels of glutathione and increased peroxidation of hepatic lipids, which are both implicated in the induction of OS (50, 51). Huang et al. (15) reported that higher blood EO levels showed an increased risk of COPD, which is mediated by inflammation and OS. Furthermore, a cross-sectional study observed that EO may elevate the risk of asthma, a condition potentially mediated by systemic inflammation (13). There have been few investigations into the relationship between EO and bone health. Further studies are required to elucidate the responsible mechanisms.

The present study has some constraints. First, due to its cross-sectional nature, the potential presence of unmeasured confounders cannot be excluded, and causal relationships cannot be inferred. Secondly, only one HbEO measurement was utilized to assess chronic exposure to EO, which may have led to exposure misclassification, as fluctuations in blood EO levels over time were not estimated. Thirdly, relying on self-generated data in the NHANES dataset may have resulted in bias, specifically, in terms of recall and reporting. Finally, DXA scans were only performed on individuals aged 8 to 59 years, excluding older ones, which limits the generalizability of the results. Further prospective cohort investigations are needed to address these limitations and verify the findings.

## Conclusion

In conclusion, the exposure to EO and the total BMD were found to be significantly negatively correlated, which the total BMD of participants with the highest quartile HbEO level was 0.01944 g/cm^2^ lower than that of participants with the lowest quartile HbEO level. However, further randomized controlled trials are necessary to determine a causal link between EO and BMD. These results could have considerable implications for stratifying the possibility of osteoporosis in the normal population.

## Data Availability

The original contributions presented in the study are included in the article/Supplementary material, further inquiries can be directed to the corresponding author.
