# Supplementary material for: Associations between blood ethylene oxide levels and bone mineral density
Source: Front Public Health. 2025 May 22;13:1561920. doi: 10.3389/fpubh.2025.1561920 (PMC12142054; doi:10.3389/fpubh.2025.1561920)
Supplement: Supplementary file 1 [file Table_1.docx]

### Supplementary Table 1. Association of HbEO with Lumbar spine BMD.

|  | Q1 | Q2 | Q3 | Q4 | P for trend |
| --- | --- | --- | --- | --- | --- |
| β (95% CI) |  |  |  |  |  |
| Model II | Ref | 0.01207(-0.00996, 0.03410) | -0.00919(-0.03009, 0.01170) | -0.02148(-0.04595, 0.00300) | 0.048 |

Abbreviations: HbEO: hemoglobin-bound ethylene oxide; BMI: body mass index; BMD: Bone mineral density; COPD, chronic obstructive pulmonary disease; CKD, chronic kidney disease; DM, diabetes mellitus; IFG, impaired fasting glucose; IGT, impaired glucose tolerance; HbA1c, glycated hemoglobin A1c; ALT, alanine aminotransferase; AST, aspartate aminotransferase; MET, metabolic equivalent task.

Model 2: Adjusted for age, race, gender, BMI, DM, hypertension, CKD, asthma, MET, drinking status, ALT, AST, hemoglobin, and HbA1c, dietary calcium intake, vitamin D levels.

Q1 ≤22.65; 22.65 <Q2 ≤32.7; 32.7< Q3≤92.77; Q4> 92.77.
