# Supplementary material for: Associations between blood ethylene oxide levels and bone mineral density
Source: Front Public Health. 2025 May 22;13:1561920. doi: 10.3389/fpubh.2025.1561920 (PMC12142054; doi:10.3389/fpubh.2025.1561920)
Supplement: Supplementary file 3 [file Table_3.docx]

### Supplementary Table 3. Association of HbEO with TBMD (continuous).

|  | β (95% CI) | P |
| --- | --- | --- |
|  |  |  |
| Model II | -0.00003(-0.00007, 0.00000) | 0.04983 |

Abbreviations: HbEO: hemoglobin-bound ethylene oxide; BMI: body mass index; TBMD: Total bone mineral density; COPD, chronic obstructive pulmonary disease; CKD, chronic kidney disease; DM, diabetes mellitus; IFG, impaired fasting glucose; IGT, impaired glucose tolerance; HbA1c, glycated hemoglobin A1c; ALT, alanine aminotransferase; AST, aspartate aminotransferase; MET, metabolic equivalent task.

Model 2: Adjusted for age, race, gender, BMI, DM, hypertension, CKD, asthma, MET, drinking status, ALT, AST, hemoglobin, and HbA1c, dietary calcium intake, vitamin D levels.
