# Supplementary material for: Associations between blood ethylene oxide levels and bone mineral density
Source: Front Public Health. 2025 May 22;13:1561920. doi: 10.3389/fpubh.2025.1561920 (PMC12142054; doi:10.3389/fpubh.2025.1561920)
Supplement: Supplementary file 4 [file Table_4.docx]

### Supplementary Table 4. Variance inflation factor (VIF) analyses of model 2.

|  | GVIF |
| --- | --- |
| Age | 1.725425 |
| Race/ethnicity | 1.525172 |
| BMI | 1.284556 |
| Sex | 1.840603 |
| HbA1c | 1.899926 |
| Alt | 3.882994 |
| Ast | 3.660088 |
| Hemoglobin | 1.910220 |
| Serum vitamin D level | 1.284422 |
| dietary calcium intake | 1.079259 |
| Drinks | 1.104473 |
| CKD | 1.049445 |
| Asthma | 1.035832 |
| Hypertension | 1.250903 |
| Diabetes mellitus | 1.840266 |
| MET(met·min/week) | 1.109174 |
| EOHQ | 1.347543 |

Abbreviations: HbEO: hemoglobin-bound ethylene oxide; BMI: body mass index; BMD: Bone mineral density; COPD, chronic obstructive pulmonary disease; CKD, chronic kidney disease; DM, diabetes mellitus; IFG, impaired fasting glucose; IGT, impaired glucose tolerance; HbA1c, glycated hemoglobin A1c; ALT, alanine aminotransferase; AST, aspartate aminotransferase; MET, metabolic equivalent task.
